# A Rare Case of Right Retrocaval Ureter with Duplication of Infrarenal IVC

**DOI:** 10.1155/2014/345712

**Published:** 2014-11-13

**Authors:** Anees Dudekula, Sonali D. Prabhu

**Affiliations:** Department of Radiodiagnosis, Kasturba Medical College and Other Affiliated Hospitals, Manipal University, Attavar, Mangalore, Karnataka 575 001, India

## Abstract

Retrocaval ureter, also known as circumcaval ureter, is a rare congenital anomaly which commonly presents with loin pain in middle age group. Here, ureter passes between the inferior vena cava (IVC) and psoas muscle and gets compressed. Duplication of IVC is another rare congenital anomaly in the development of IVC. We present a case of a 49-year-old male who presented with loin pain and upon thorough investigation was found to have retrocaval ureter along with duplication of the infrarenal IVC. We bring forward this rare type of combination of two congenital malformations.

## 1. Introduction

Retrocaval ureter, also known as circumcaval ureter, is a rare congenital anomaly which usually presents in the 3rd to the 4th decade of life. The commonest presenting complaint is loin pain caused by hydronephrosis due to compression of the right ureter between the IVC and psoas muscle as ureter courses posterior to IVC. Retrocaval ureter is commonly seen on right side and rarely on left side if associated with situs inversus or IVC anomalies-left IVC or rarely duplicated IVC.

Duplication of IVC is a very rare congenital anomaly in the development of IVC which can go totally undetected in the entire life. Duplication of IVC is most commonly detected on autopsy specimens or may be incidentally diagnosed if associated with other congenital malformations like renal anomalies, retrocaval ureter, venous thromboembolism, and so forth. Even though many cases of right retrocaval ureter are reported, only a few cases of retrocaval ureter with duplication of IVC have been reported. Duplicated IVC can present with either unilateral or bilateral retrocaval ureter. We are reporting a case of right retrocaval ureter with IVC duplication.

## 2. Case Report

A 49-year-old male patient presented with complaints of recurrent right loin pain since 3 years. On clinical examination, patient had minimal tenderness in right loin on deep palpation. Results of urine analysis were within normal limits. Patient was referred for sonography.

**Figure 1:**
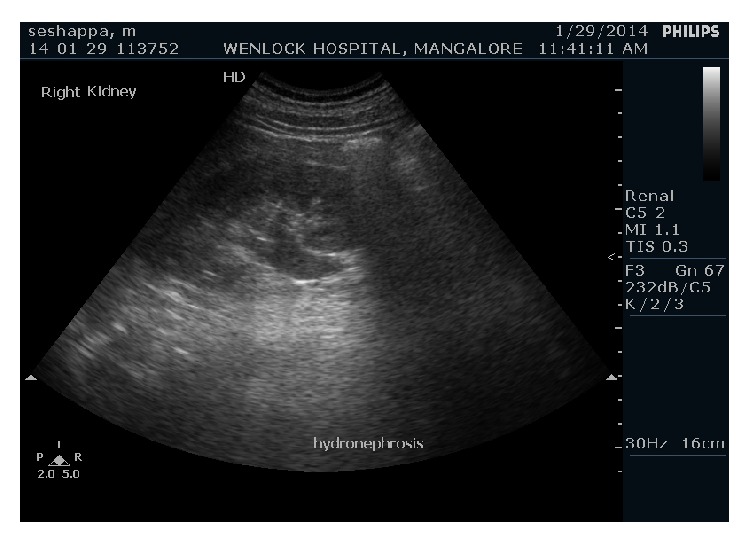
Grey scale ultrasound examination of abdomen shows dilated right renal ureter, renal pelvis, and right hydronephrosis.

**Figure 2:**
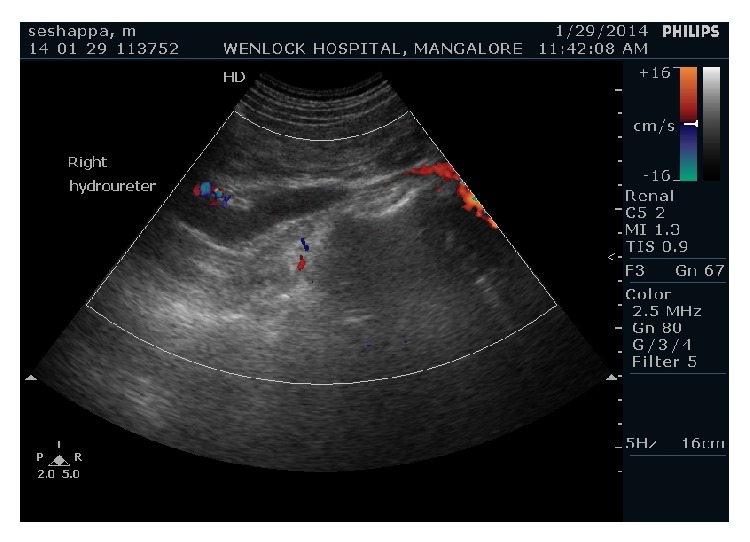
Ultrasound examination with Doppler shows abrupt narrowing of the dilated right ureter at the level of right iliac vessels.

Ultrasound of abdomen revealed mild hydronephrosis of right kidney with dilated right upper ureter and abrupt narrowing at the level of crossing of right iliac vessels. Lower ureter at the level of urinary bladder was of normal calibre. There was no evidence of any calculus in the right ureter or vesicoureteric junction (Figures 1 and 2).

**Figure 3:**
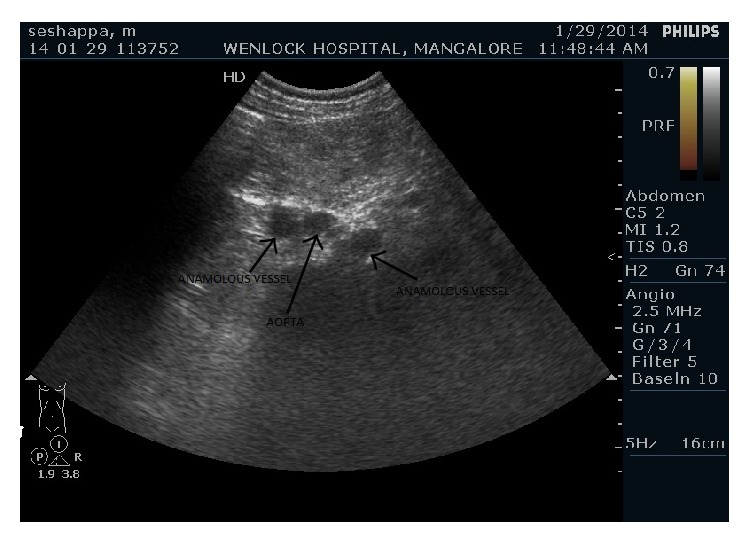
Grey scale ultrasound examination of abdomen shows evidence of anomalous vessel on either side of the lower abdominal aorta.

Gray scale imaging also revealed evidence of vessel on either side of the lower abdominal aorta. Color Doppler sonography with spectral examination of both of these vessels revealed the flow to be of low resistance continuous flow pattern consistent with that of venous flow (Figure 3). Probable diagnosis of aberrant venous channel to the left of aorta was made.

Due to presence of right renal hydronephrosis with abrupt narrowing of right ureter in midsegment and presence of anomalous vascular pattern, computed tomography (CT) with delayed excretory scanning was advised for further evaluation.

**Figure 4:**
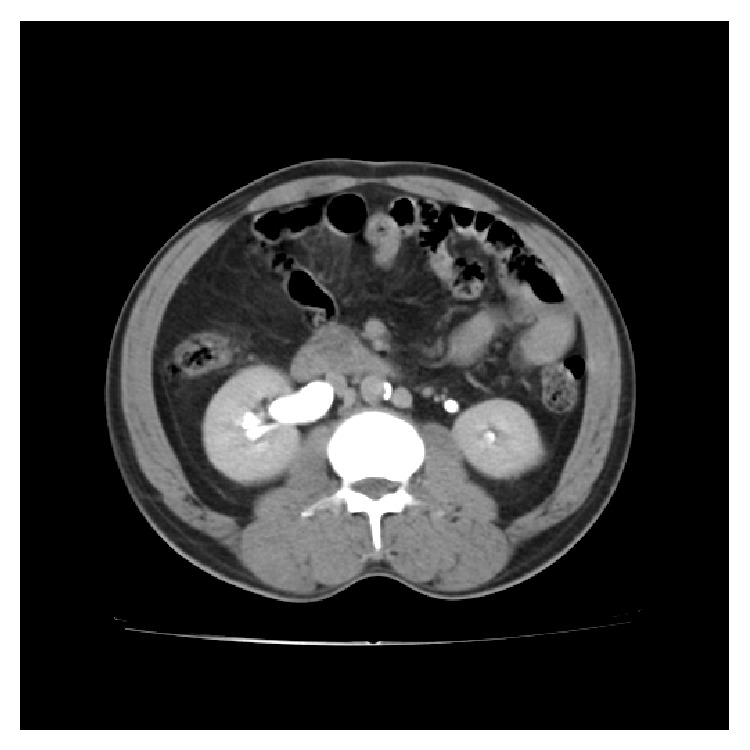
CECT delayed phase shows right hydronephrosis with dilated upper ureter.

**Figure 5:**
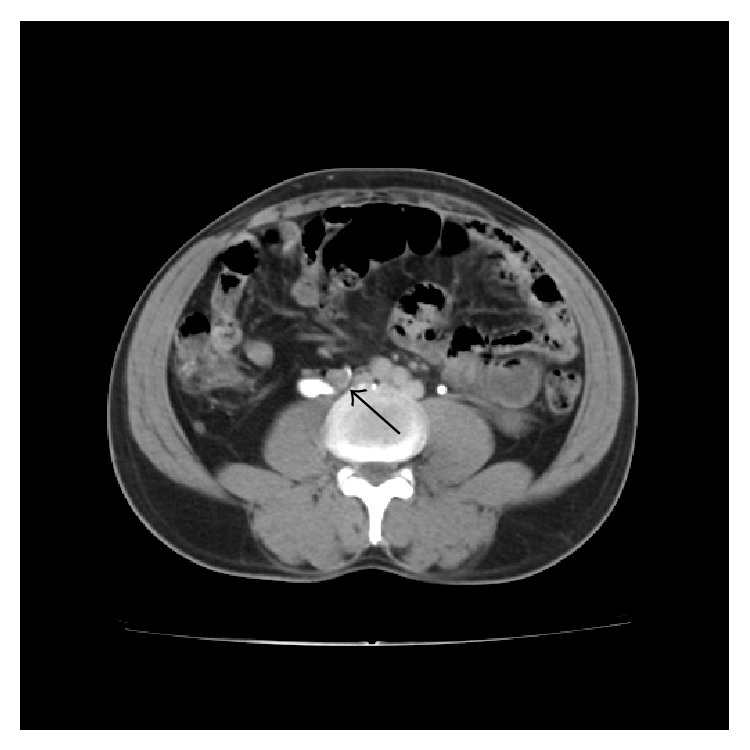
CECT delayed phase shows the right ureter traversing behind the right IVC (arrow) with extrinsic compression at that site causing dilatation of the proximal ureter.

**Figure 6:**
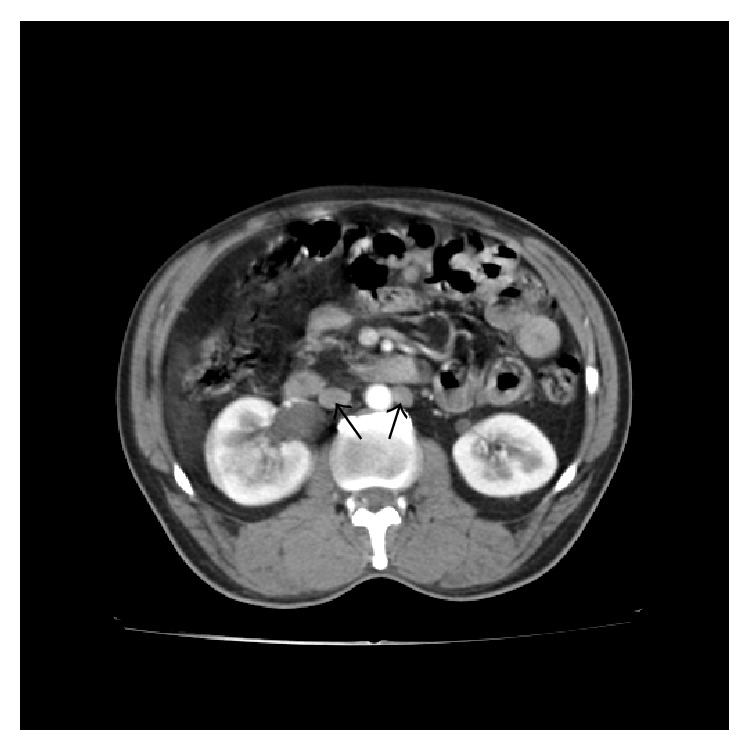
CECT shows duplicated IVC (arrow) on either side of the abdominal aorta.

CT of abdomen showed mild hydronephrosis of the right kidney with dilated upper ureter. Multiplanar reconstruction showed dilated upper ureter with retrocaval course of right ureter forming a typical fish hook or reverse J appearance at the L3-L4 intervertebral level (Figure 4). The right ureter was seen coursing posterior to the inferior vena cava (Figure 5). There was an anomalous vessel to the left of infrarenal aorta contiguous with the left common iliac vein and it received the venous return from left lower limb. This vessel joined the left renal vein and crossed anterior to the aorta draining into the right IVC (Figure 6).

## 3. Discussion

The embryogenesis of the IVC is a complex process of development. A proper knowledge about the sequence of appearance, anastomosis, and regression of the different paired embryonic veins in the development of IVC is necessary for understanding the rare IVC anomalies such as those presented in this case.

In embryology, the IVC develops from a plexus of fetal veins. The posterior cardinal and supracardinal veins lie dorsally and the subcardinal veins lie ventrally. The left supracardinal veins and the lumbar portion of the right posterior cardinal vein atrophy. The subcardinal veins become the internal spermatic veins. The definite right-sided inferior vena cava forms from the right supracardinal vein [1].

Duplication of the IVC results from persistence of the inferior portion of the left supracardinal vein. This anomaly is identified in 2% to 3% of autopsy specimens, but it is observed much less frequently (0.3%) on imaging studies. Each iliac vein drains into its corresponding vena cava. The left IVC usually joins the right IVC at the level of the left renal vein [2].

**Figure 7:**
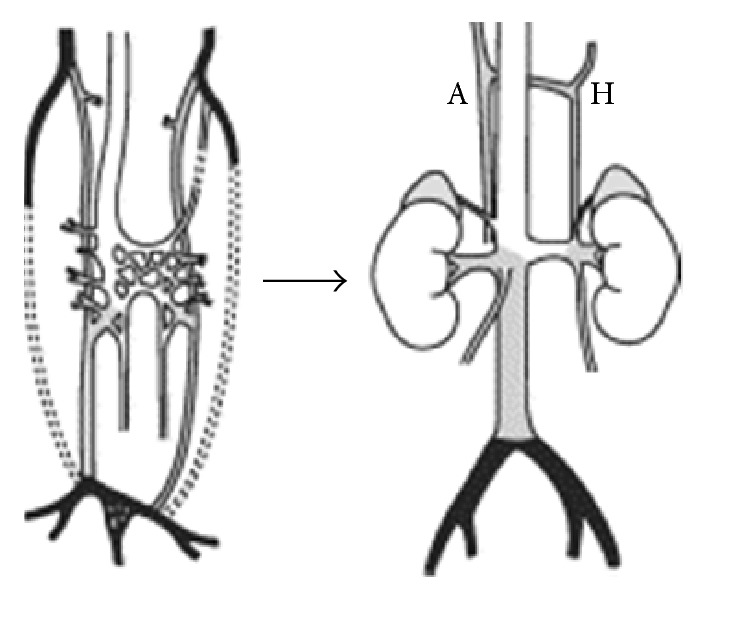
Embryologic development of the inferior vena cava. Shading indicates the posterior cardinal origin (black), supracardinal origin (gray), and subcardinal origin (white). A: azygos vein; H: hemiazygos vein (adapted from Lundell C, Kadir S: Inferior vena cava and spinal veins. In: Kadir S, ed: Atlas of Normal and Variant Angiographic Anatomy. Philadelphia, WB Saunders, 1991:187).

Retrocaval ureter occurs in 1 in 1000 population [3]. Most cases are asymptomatic and are detected only during routine radiologic imaging [4]. There is male to female ratio of 3 : 1 [4]. It occurs more commonly on the right side compared to the left side and is usually associated with other inferior vena cava (IVC) anomalies [5, 6]. Most of the patients with this anomaly usually present with obstructive symptoms because of the compression of the ureter. If the right subcardinal vein in the lumbar portion fails to atrophy and forms the infrarenal IVC, the ureter is trapped dorsally into it and forms retrocaval ureter (Figure 7) [1].

Two types of retrocaval ureter are frequently encountered—Type I and Type II. In Type I, the ureter crosses behind the inferior vena cava at the level of the third lumbar vertebra and it has a “J” or fish-hook shape at the point of obstruction. Marked hydronephrosis is seen in 50% of patients. In the less common Type II, the crossover occurs higher at the level of the renal pelvis. Mild hydronephrosis is usually seen in the majority of these patients. Both types are frequently detected in the 2nd, 3rd, and 4th decades of life [7].

Our case is unique as two rare anomalies have occurred concurrently. The importance of diagnosing duplication of IVC has been well documented. Such awareness will prevent problems when planning repair of aortic aneurysm, placement of IVC filter in lower limb venous thrombosis, for evaluation of donor in renal transplant and during surgeries like renal transplantation, nephrectomy and adrenalectomy. It will aid in evaluating retroperitoneal masses and adenopathy and avoid accidental ligation which can lead to torrential bleed and death [8, 9].

Radiological examinations including intravenous urography, sonography, CT, and MRI scanning are techniques which will aid in coming to a diagnosis. In summary, a rare case of right retrocaval ureter associated with duplication of the IVC is reported. The diagnosis of retrocaval ureter is important as resolution of symptoms is possible with surgical correction. Also prior information about presence of associated IVC anomalies can prevent untoward surgical complications.
